# Information security education: a thematic trend analysis

**DOI:** 10.12688/f1000research.159828.1

**Published:** 2025-01-02

**Authors:** Paula Andrea Rodríguez-Correa, Alejandro Valencia-Arias, Ezequiel Martínez Rojas, Aarón Oré León, Ray Harvey Mellin Rubio, Manuel Humberto Vásquez Coronado, Jesus Alberto Jimenez Garcia

**Affiliations:** 1 Institución Universitaria Escolme, Medellín, Antioquia, 050010, Colombia; 2 Universidad Senor de Sipan, Chiclayo, Lambayeque, Peru; 3 Universidad Arturo Prat, Iquique, Tarapacá Region, Chile; 4 Universidad Ricardo Palma, Santiago de Surco, Lima, Peru; 5 Universidad Católica de Trujillo Benedicto XVI, Trujillo, Peru

**Keywords:** Information security, cybersecurity, data protection, information security training, higher education

## Abstract

The evolution of information technologies has led to a significant increase in information security risks, underscoring the urgent need for professional education in this field to safeguard organizational data. Publications on this topic highlight the necessity of educating individuals about data security, and their number has grown in recent years. This study aims to identify thematic trends in information security education through a bibliometric analysis based on five research questions, following PRISMA guidelines. Ninety-nine documents from Scopus and Web of Science were analyzed, revealing a quadratic growth, with 2023 as the year of greatest research activity. Prominent contributors such as Von Solms, Safa, and Furnell stand out, along with the journal Computers & Security and the significant influence of the United States in this field. The potential is identified in the following areas: information security culture, media, information security regulations, higher education institutions, information security management, and cybersecurity. The study also identifies gaps and proposes a research agenda to address these gaps.

## 1. Introduction

As information technologies advance, information security risks also increase. Consequently, companies are increasingly concerned about cyber-attacks ( Safa et al., 2015). Thus, information security has become one of the most important and debated topics among experts. Actions are being taken by implementing organizational information security procedures and policies, encouraging information security-conscious behavior, and promoting the sharing of information security knowledge ( Safa et al., 2018).

Given the rapid technological evolution, continuous evaluation and constant innovation to improve security systems, such as multi-layer firewalls, have become essential. According to Torten et al. ( Torten et al., 2018), cyberattacks often target the installation of ransomware, intellectual property infringement, theft of medical records, unauthorized banking transactions, and credit card misuse. Consequently, companies worldwide have placed a high priority on information systems risk management ( Flores & Ekstedt, 2016).

Given the growing importance of protecting information systems within organizations, there has been an increased focus on developing human talent in cybersecurity in recent years as a key component of information technology education. This focus is reflected in both core and advanced curricula ( Rowe et al., 2011). In addition, higher education plays a key role in society, particularly in terms of research, development, and education. Consequently, faculties are establishing their own Information Technology (IT) networks designed to support research, development, and teaching activities in the field of information security ( Ulven & Wangen, 2021).

Therefore, several literature reviews have focused on investigating the fundamental knowledge, skills, and capabilities required by cybersecurity professionals ( Jones et al., 2018), as well as analyzing information security curricula ( Parrish et al., 2018). These studies have highlighted that information security has emerged as a crucial area of learning that demands specific professional education, particularly in areas such as secure programming, network security, and offensive security ( Švábenský et al., 2020).

Information security education and training are critical for preparing both current and future IT professionals to adequately and effectively address real-world security risks and incidents ( Beuran et al., 2016). Consequently, universities recognize the importance of staying abreast of cyber threats and risks to provide students with up-to-date cybersecurity tools and knowledge through innovative methodologies ( Cheung et al., 2011).

Thus, it has been evidenced that in the current era, where information is an invaluable asset and technology drives most human activities, information security has become a paramount concern ( Burov et al., 2020). This concern is compounded by the increasing sophistication of cyber threats and the proliferation of cyber-attacks in both business and personal domains ( Khaleefah & Al-Mashhadi, 2024). Therefore, the growing need for trained information security professionals is evident, making university programs dedicated to information security education essential ( AlDaajeh et al., 2022).

Based on the background of the literature, the objective of this research is established: to identify the thematic trends in information security education. To this end, the following research questions are formulated:

• Who are the research actors with the greatest impact on the field?
• What is the thematic evolution of the information security education phenomenon in recent years?
• What are the research clusters related to the phenomenon under study?
• What are the most frequent and current topics within the information security education phenomenon?
• What are the most promising topics for future research on the phenomenon under study?

## 2. Methods

A bibliometric analysis was used, a technique that allows for evaluating the quality and quantity of published scientific literature, as well as studying research trends and various citation analyses within a given field. It is considered a useful and productive tool for determining research trends across different disciplines ( Buber & Koseoglu, 2022). To ensure data quality, the parameters of the PRISMA 2020 guidelines for systematic reviews and meta-analyses were followed. The PRISMA guidelines establish protocols for proper literature review, including a four-phase flowchart and a 27-item checklist ( Selçuk, 2019). The phases followed in this study are detailed below.

### 2.1 Eligibility criteria

One of the first steps to meet the criteria of the PRISMA statement is to establish the inclusion and exclusion criteria used in the selection of documents ( Moraga & Cartes-Velásquez, 2015). For inclusion, a search was conducted using the following keywords: “information,” “security,” “education,” and “training.” As for exclusion criteria, conference proceedings were omitted, as they typically represent very recent studies that have not yet been consolidated as relevant research in the field addressed by this study. Additionally, documents with indexing errors were excluded.

### 2.2 Information sources

The two most commonly used databases for this type of analysis were selected: Scopus and Web of Science. The choice of these databases is based on their significant differences in terms of coverage, accessibility, updating, and the number of citations retrieved ( Manriquez et al., 2015).

### 2.3 Search strategy

As part of the search strategy, a search equation was applied in both databases. The AND operators were used to include the terms “information” and “security” in the search. Additionally, OR was used to broaden the search to include the fields of "education" or "training." The following search equations were applied:

In Scopus: TITLE (information AND security AND (education OR formation))

In Web of Science: TI= (information AND security AND (education OR formation))

### 2.4 Selection process

**Figure 1.**
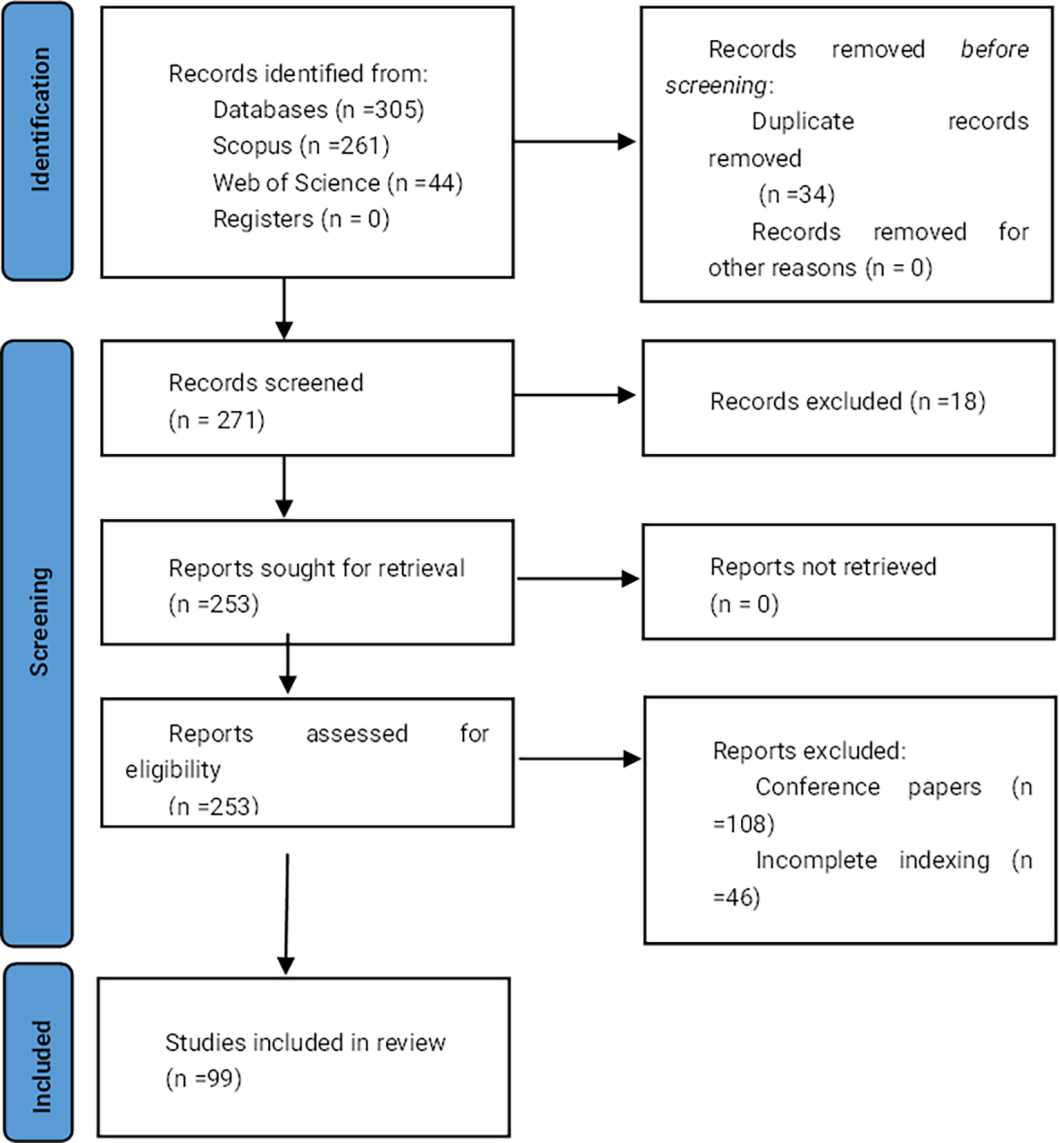
PRISMA flow chart. Own elaboration based on Scopus and Web of Science.

Once the search equations were applied in the databases, 261 records were obtained from Scopus and 44 from Web of Science, totaling 305 records. Subsequently, duplicates were removed, resulting in 271 documents that were then subjected to inclusion and exclusion criteria. After applying these eligibility criteria, 99 documents were selected for analysis. Figure 1 displays the flowchart of the document selection process.

## 3. Results

**Figure 2.**
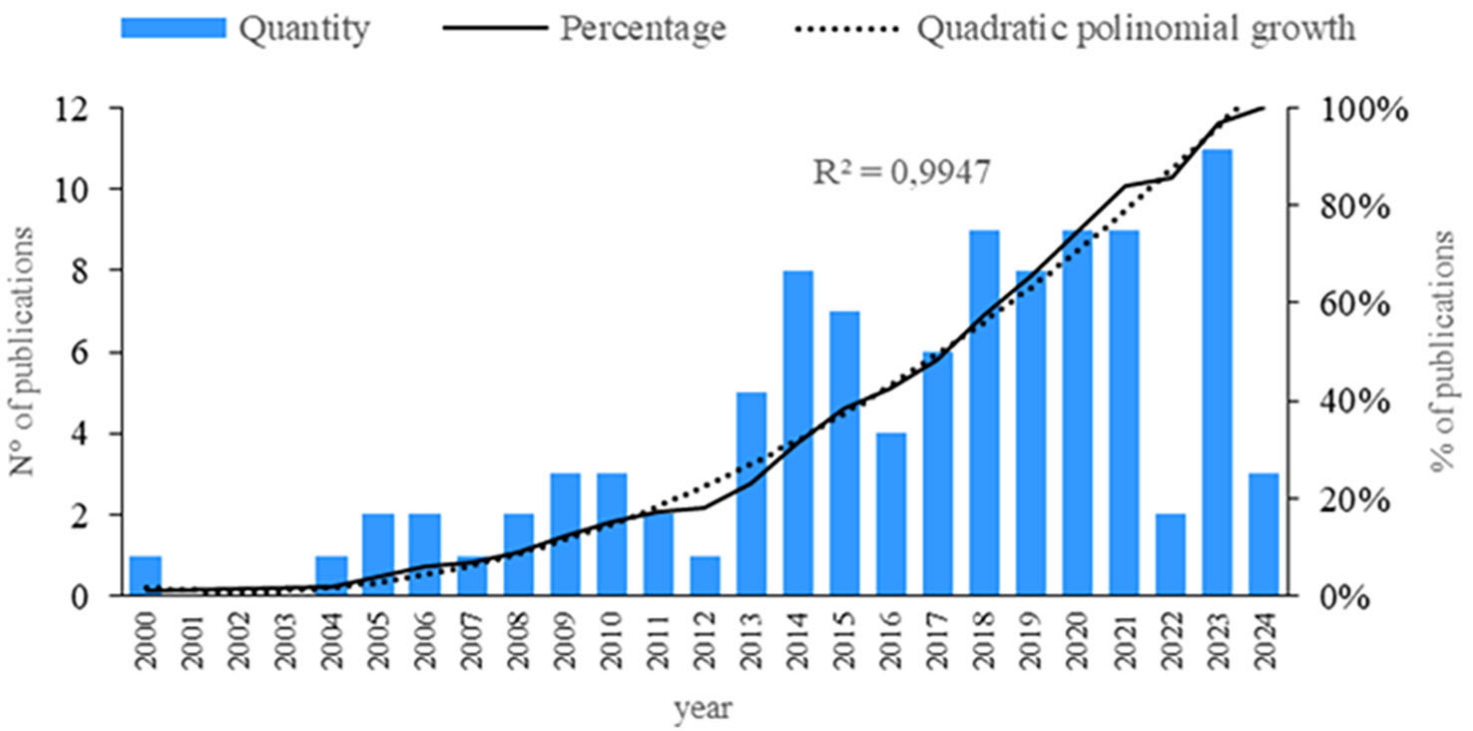
Publications by year. Own elaboration based on Scopus and Web of Science.

First, an analysis of publications by year is conducted. As shown in Figure 2, a steady increase has been observed in recent years regarding the subject of this research. However, a decrease is noted in 2022, followed by an increase in 2023. Therefore, since 2013, there has been a continuous rise in the number of publications. Additionally, an analysis of quantity, quality, and structural indicators is performed.

### 3.1 Analysis of the most important research actors

**Figure 3.**
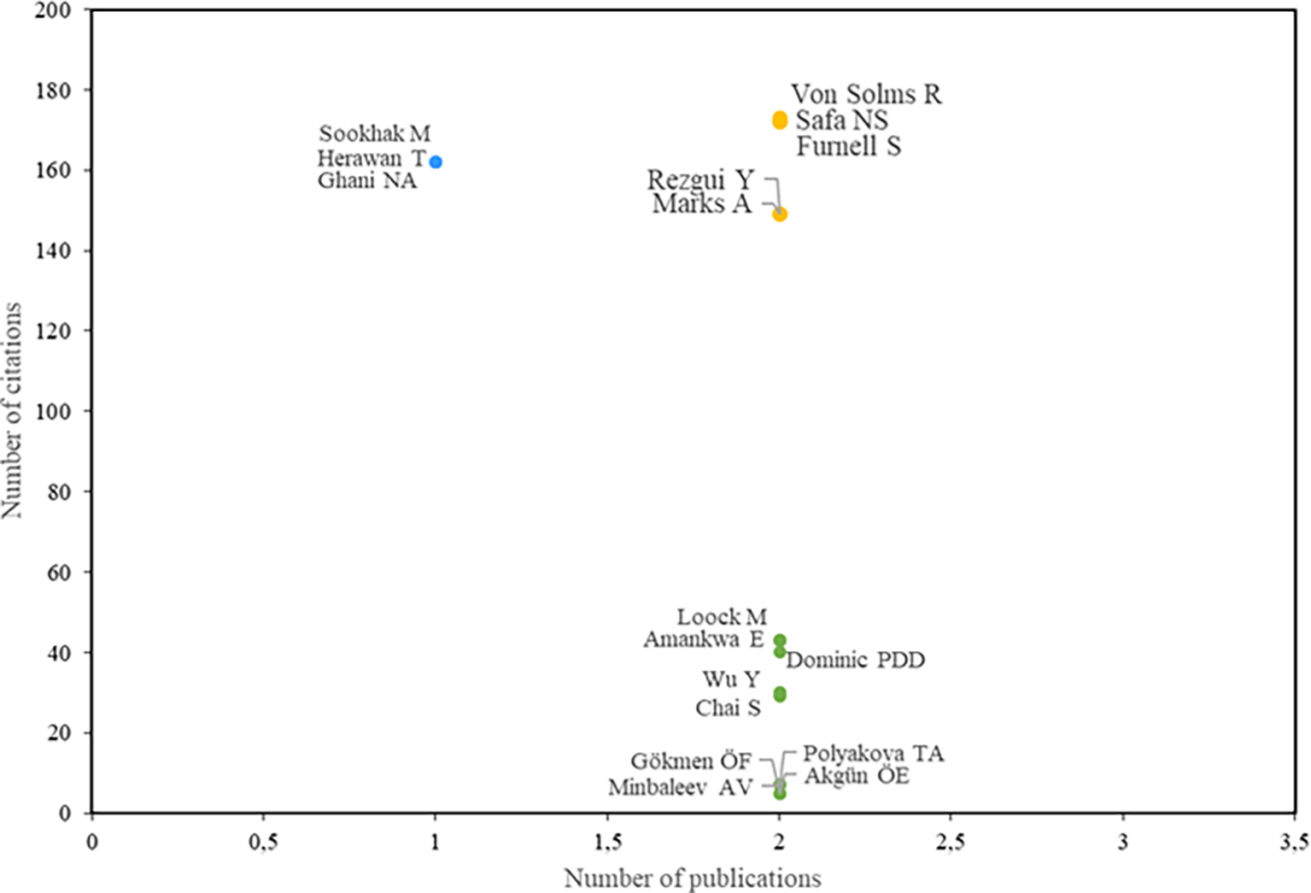
Most relevant authors. Own elaboration based on Scopus and Web of Science.

As stated in the first research question, the aim is to identify the most relevant research actors. Figure 3 displays the most influential authors. The blue color indicates the authors with the highest number of citations, while the green color represents the authors with the highest number of publications. Authors highlighted in yellow are those with both the highest number of publications and the highest number of citations.

In this context, authors Von Solms R., Safa N.S., and Furnell S. stand out as significant contributors to the field. In 2015, they conducted research on the education of information security-conscious behaviors in organizations. This study evaluated various factors, including information security awareness, organizational information security policies, information security experience, and involvement, attitudes toward information security, subjective norms, threat assessment, information security self-efficacy, and user capability ( Safa et al., 2015).

Rezgui Y. and Marks A. have also made significant contributions to this area of study. Their research focuses on the determinants of information security awareness among university personnel, including those involved in information systems decision-making, within the specific context of a developing country. They identified that variables such as conscientiousness, cultural norms and beliefs, and social dynamics significantly shape the attitudes and behaviors of academic staff, both in their general professional conduct and their awareness of information security practices ( Rezgui & Marks, 2008).

**Figure 4.**
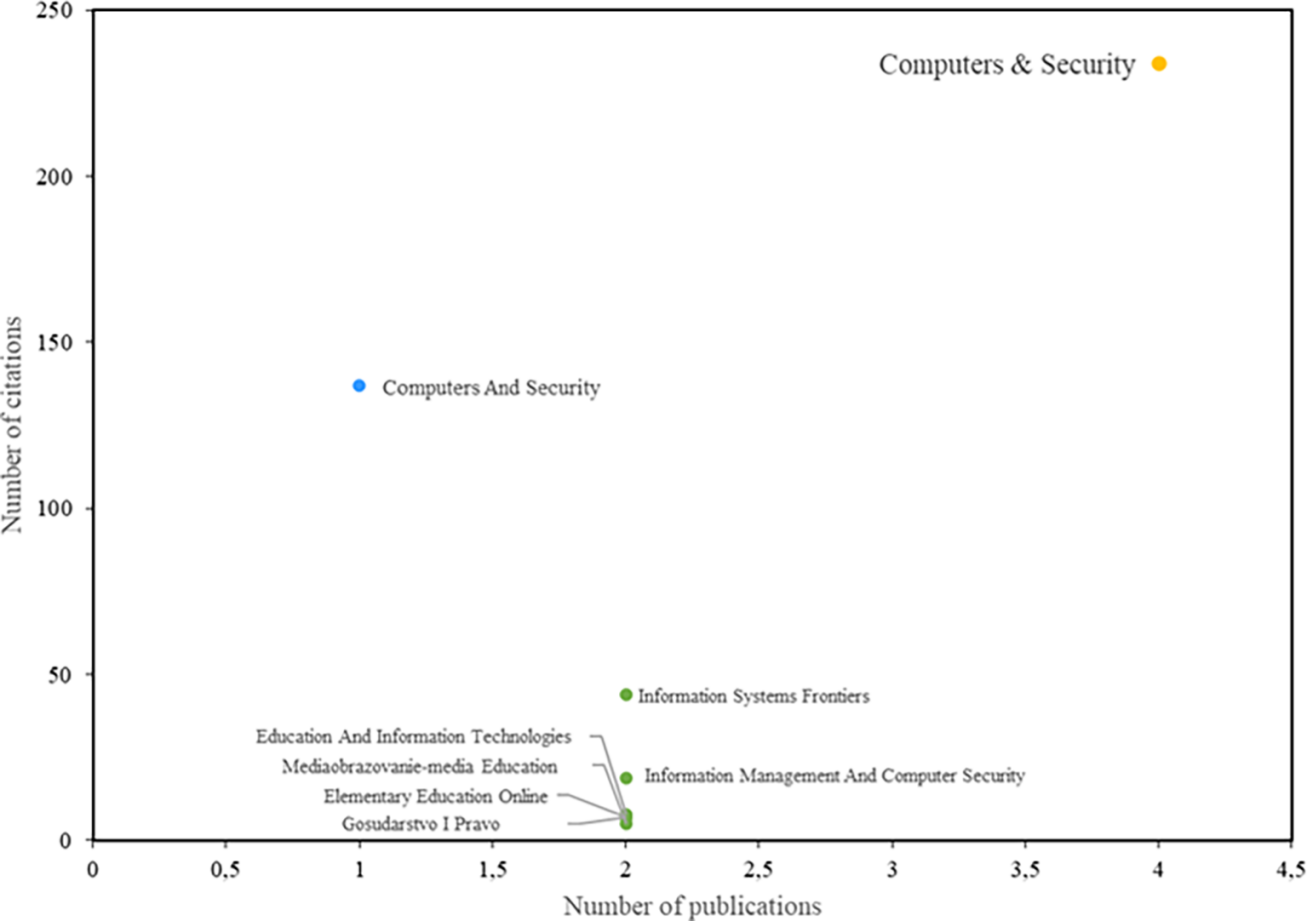
Most relevant journals. Own elaboration based on Scopus and Web of Science.

Figure 4 shows the journals with the greatest impact on the field of study. Among them, the journal Computers & Security stands out, having both the highest number of publications and the highest number of citations. Notably, the study by Safa et al. ( Safa et al., 2015) has 226 citations and is recognized as one of the most relevant publications in this field. Additionally, the study by Rezgui and Marks ( Rezgui & Marks, 2008) also warrants special attention, as it aligns with the trends identified in this research and has 138 citations.

Additionally, the study by Rajab and Eydgahi ( Rajab & Eydgahi, 2019), which has received 53 citations, is notable for presenting a theoretical framework that encompasses various behavioral theories. This framework focuses on assessing factors such as perceived vulnerability, response effectiveness, response cost, and information security compliance intentions in higher education, making it a significant contribution to the field. Likewise, the study conducted by Bongiovanni ( Bongiovanni, 2019) deserves attention, as it currently has 44 citations and focuses on literature related to information security management in higher education.

**Figure 5.**
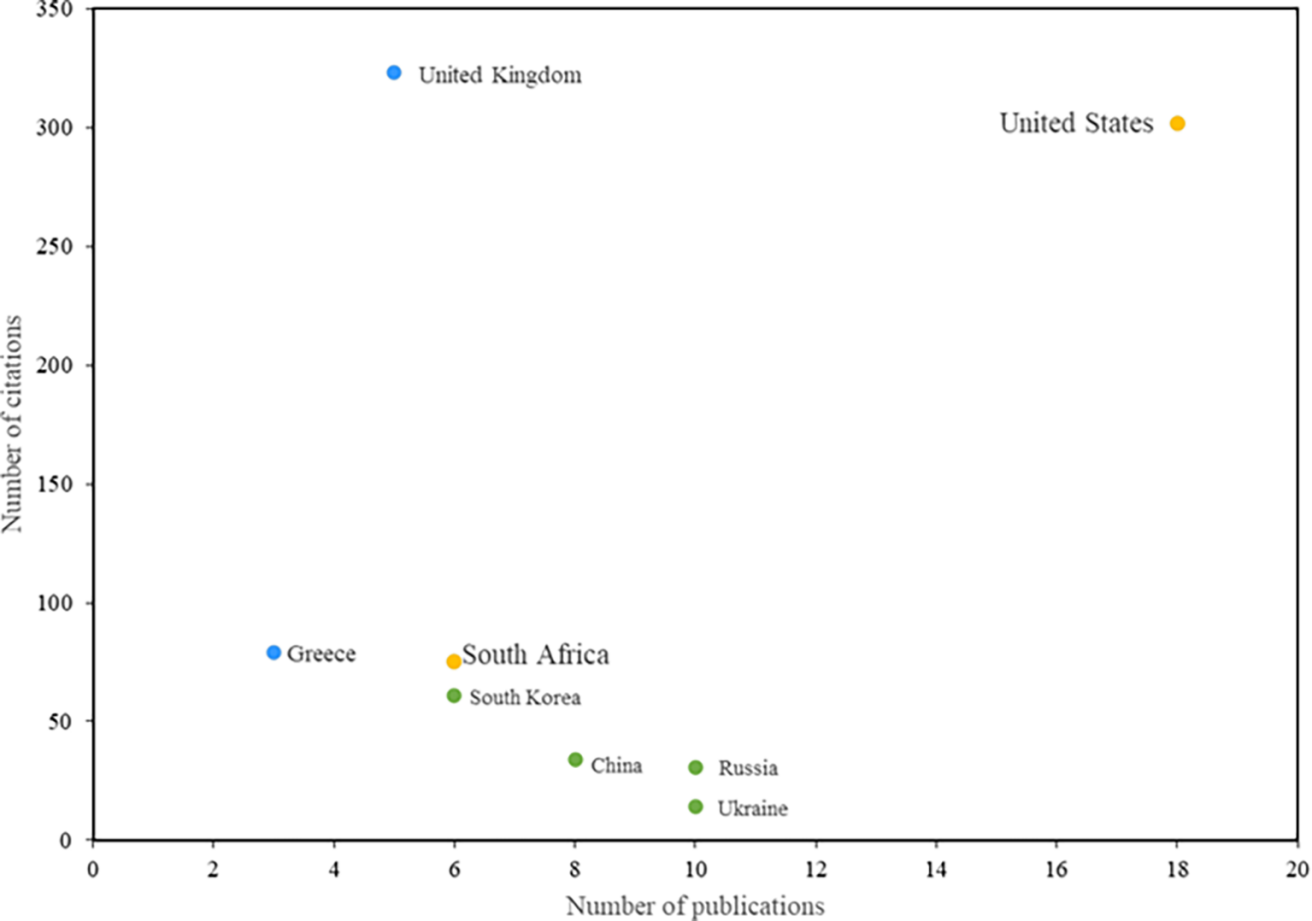
Most relevant countries. Own elaboration based on Scopus and Web of Science.

The countries that have had the greatest impact on research in information security education are presented below (see Figure 5). The United States stands out in the first place, both in terms of the number of citations and the number of publications related to this topic. Notable among these is the study by Rowe et al. ( Rowe et al., 2011), which analyzes the crucial role of cybersecurity in IT education and argues for the integration of this topic into IT programs. Also noteworthy is the study by Conklin ( Conklin, 2006), conducted in the United States, which proposes an active learning solution for a capstone course designed to enhance students’ competencies in cyberdefense and information security education.

In addition, South Africa stands out due to the significant contributions of the study by Safa et al. ( Safa et al., 2015), which has made a notable impact on the field of information security. Also highlighted is a study that provides operational definitions for information security education, information security training, and information security awareness. This research aims to help institutions determine when it is necessary to train or educate employees and when it is appropriate to implement information security awareness programs ( Amankwa et al., 2014).

### 3.2 Thematic evolution analysis

**Figure 6.**
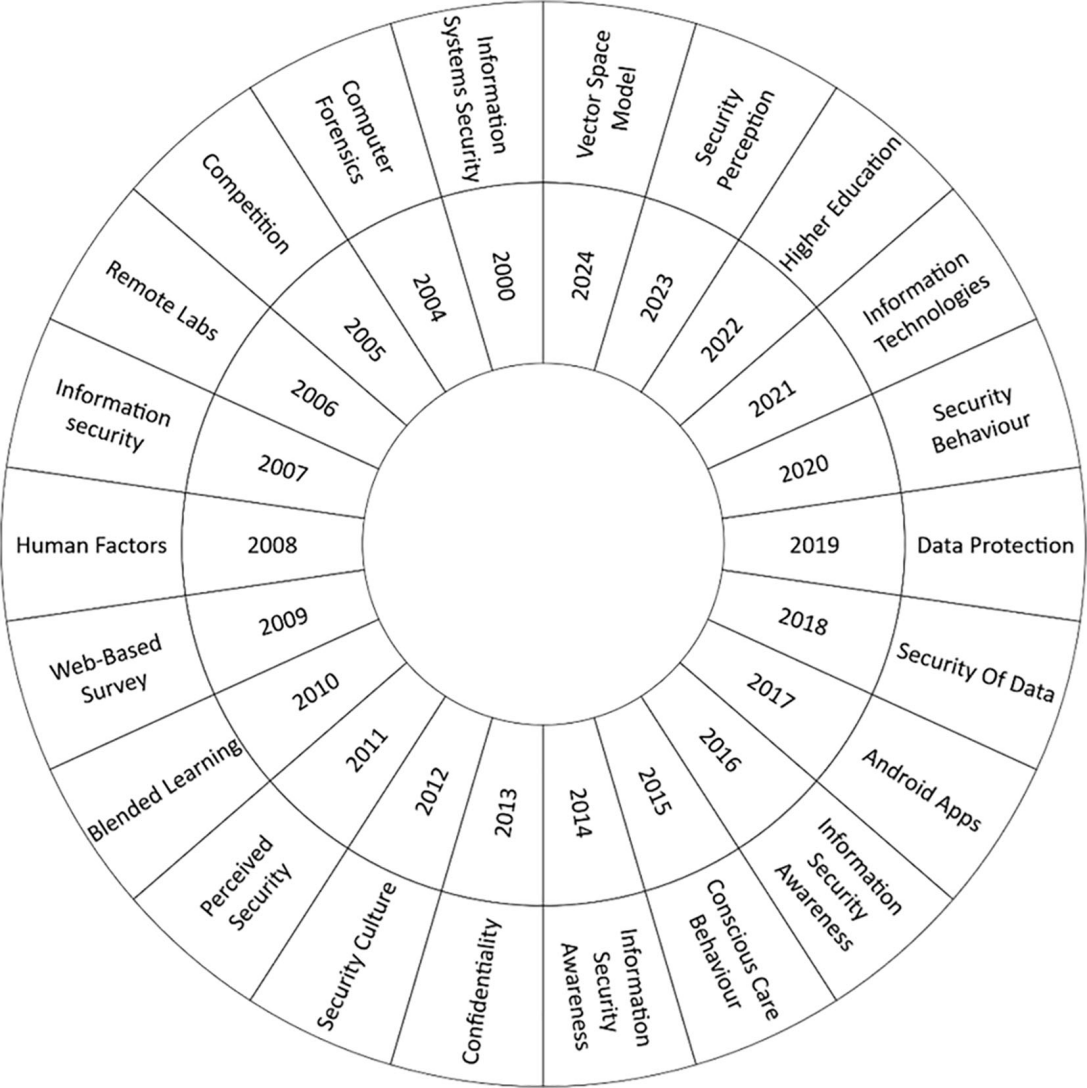
Thematic evolution. Own elaboration based on Scopus and Web of Science.

The second research question focuses on the thematic evolution of the phenomenon under study. Figure 6 presents a count of the most frequent keywords from 2000 to 2024. Recently, there has been a significant increase in interest in the topic of security perception. In this context, the work of Mohr and Walter ( Mohr & Walter, 2019) is notable, as it addresses information security education and users’ perceptions regarding the security of their personal information and online banking transactions. Another relevant topic is Higher Education, where there has been growing concern in recent years about individuals’ awareness of information security. This concern has led to the development of training programs in this area ( Wahyudiwan et al., 2017).

Another topic that has generated interest in recent years is Information Technologies, driven by the need to train personnel across various organizations and economic sectors on the security of information systems ( Katsikas, 2000). Similarly, security behavior has gained relevance during this period. With the rise in cyber-attacks affecting business performance and reputation, as well as compromising intellectual property, human factors have been identified as the weakest link in the security posture of any networked system. Consequently, studies have been conducted to analyze the relationship between threat perception and the understanding of countermeasures concerning the adoption of secure behaviors ( Torten et al., 2018).

In the last five years, data protection and data security have become highly relevant, particularly due to the increasing pressure on data storage. This rise, driven by massive data generation, has led to rapid development in the storage market. Consequently, data protection has become a critical issue for organizations ( Yang et al., 2020).

### 3.3 Thematic cluster analysis

**Figure 7.**
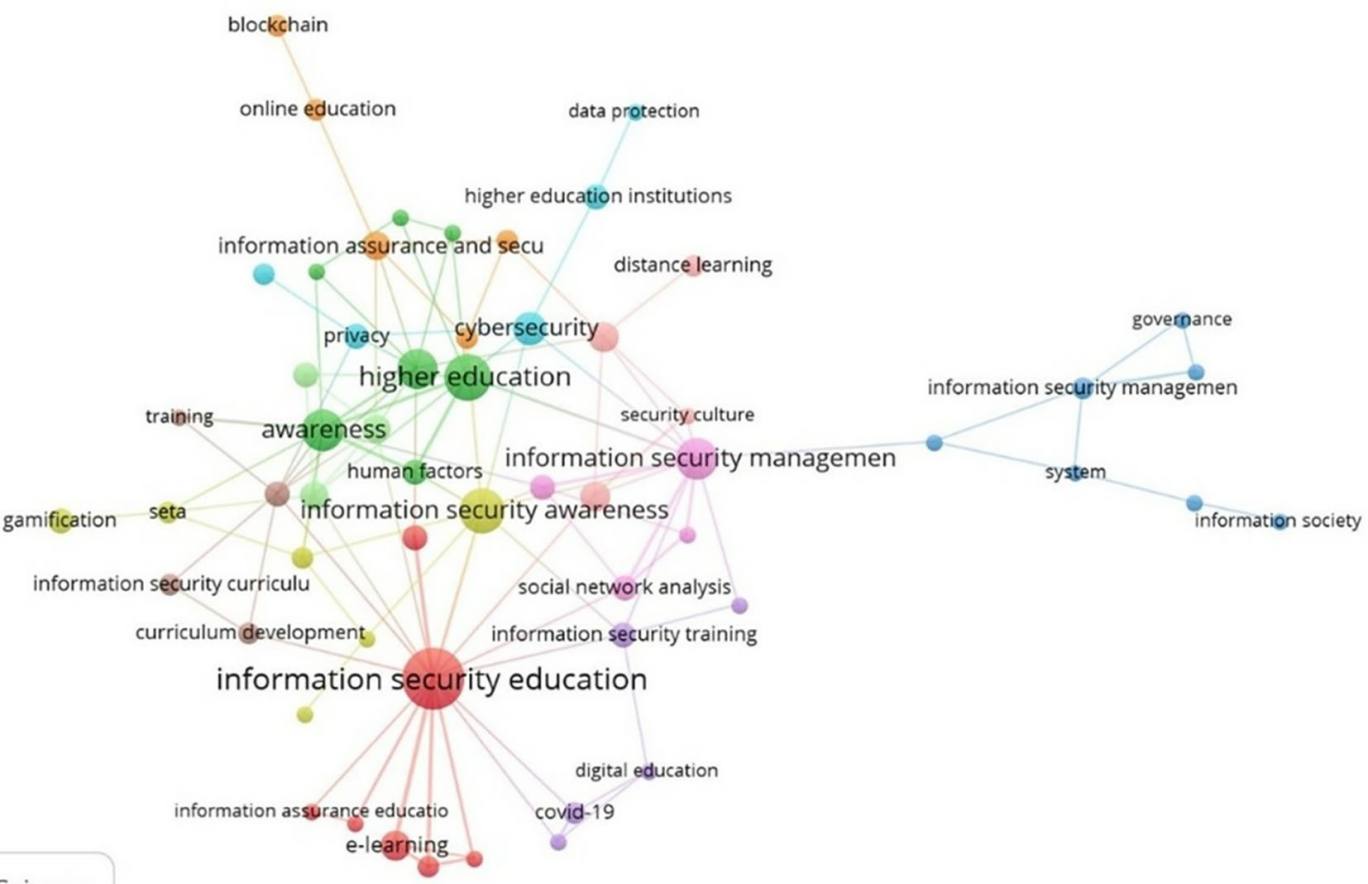
Thematic clusters. Own elaboration based on Scopus and Web of Science.

The third research question focuses on the thematic clusters present in the field of study. Figure 7 shows the keyword co-occurrence network, generated by the VOSviewer software ( Van Eck and Waltman, 2010). In this representation, several clusters in the form of nodes are identified, with the largest cluster corresponding to information security education, which is the main topic of this research. This cluster is associated with topics such as e-learning and information assurance education. In this context, researchers such as Pastor et al. ( Pastor et al., 2010) argue that the best way to enhance an individual’s response to security threats is through robust education, engaging hands-on training, and increased general awareness of information security.

An important cluster focuses on higher education, specifically addressing issues such as awareness and human factors, which, as previously highlighted, represent the weakest link in information protection. Therefore, increasing security awareness is imperative ( Brill et al., 2013). This emphasis is also reflected in the information security awareness cluster, further underscoring the need for research in this area ( Lebek et al., 2014). This cluster is associated with topics such as gamification to enhance motivation and learning outcomes. Gamification thus demonstrates significant potential for use in security awareness and training programs ( Gjertsen et al., 2017).

Generally spoken, it should look like this document. Using the styles of this document may make things easier for you, but you don’t need to use them.

In addition, the information security management cluster is highlighted due to the significant expansion of online business opportunities brought about by information technology. However, these opportunities have also introduced serious information security risks ( Soomro et al., 2016). This cluster is related to topics such as information society, systems, and governance. It encompasses areas like social network analysis, distance education, and security culture. Similarly, an information security training cluster has emerged, addressing topics such as digital education and the impact of COVID-19. Consequently, research has been conducted to analyze information security behavior and awareness among Zoom application users, particularly during the COVID-19 pandemic ( Candiwan et al., 2022).

### 3.4 Issues of currency and frequency

**Figure 8.**
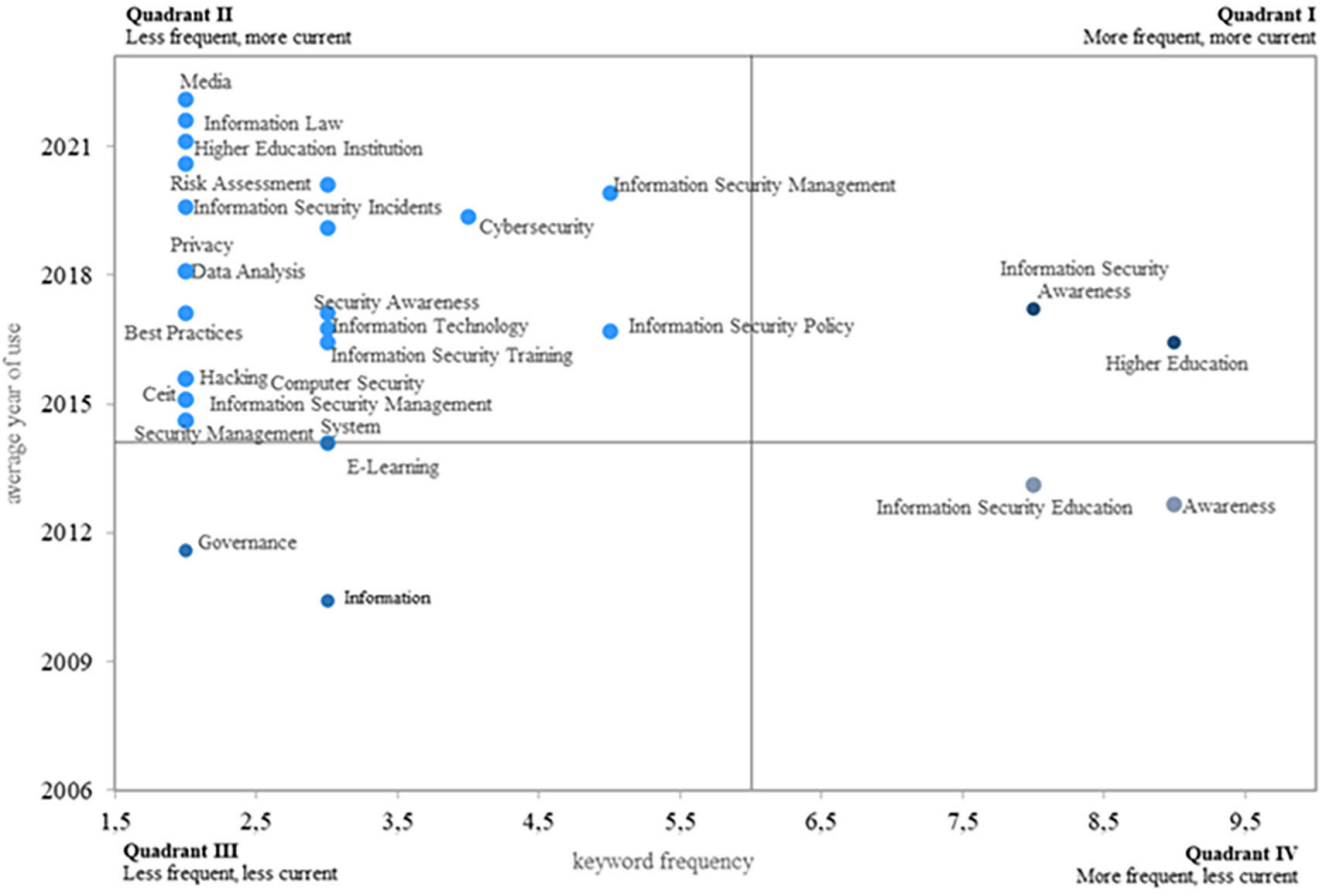
Most frequent and current topics. Own elaboration based on Scopus and Web of Science.

The fourth research question focuses on identifying the most frequent and relevant topics in the research area. Figure 8 displays four research quadrants. The first quadrant highlights the most frequent and relevant keywords, indicating topics that could guide future research on information security education. In this quadrant, the key topics are information security awareness and higher education. Notably, a study on the information security awareness of end users, including information systems decision-makers, within the context of three higher education environments is emphasized ( Rezgui & Marks, 2008).

The second quadrant highlights topics that, while less recurrent in the literature, are considered relevant, especially in recent years. Key aspects include information laws, higher education institutions (HEIs), risk assessment, cybersecurity, information security incidents, data analysis, security awareness, best practices, information technologies, information security policy, information security training, hacking, computer security, information security management, and security management. These keywords also hold significant potential for future research.

In quadrant three, less frequent and recurrent topics are identified, suggesting that they either need more momentum to become common and current or that researchers have lost interest over time. These themes include governance, information, and e-learning. Finally, quadrant four contains topics that are more frequent but less current, with evidence of a decreasing interest among researchers in recent years. Among these, awareness stands out.

### 3.5 Research agenda

**Figure 9.**
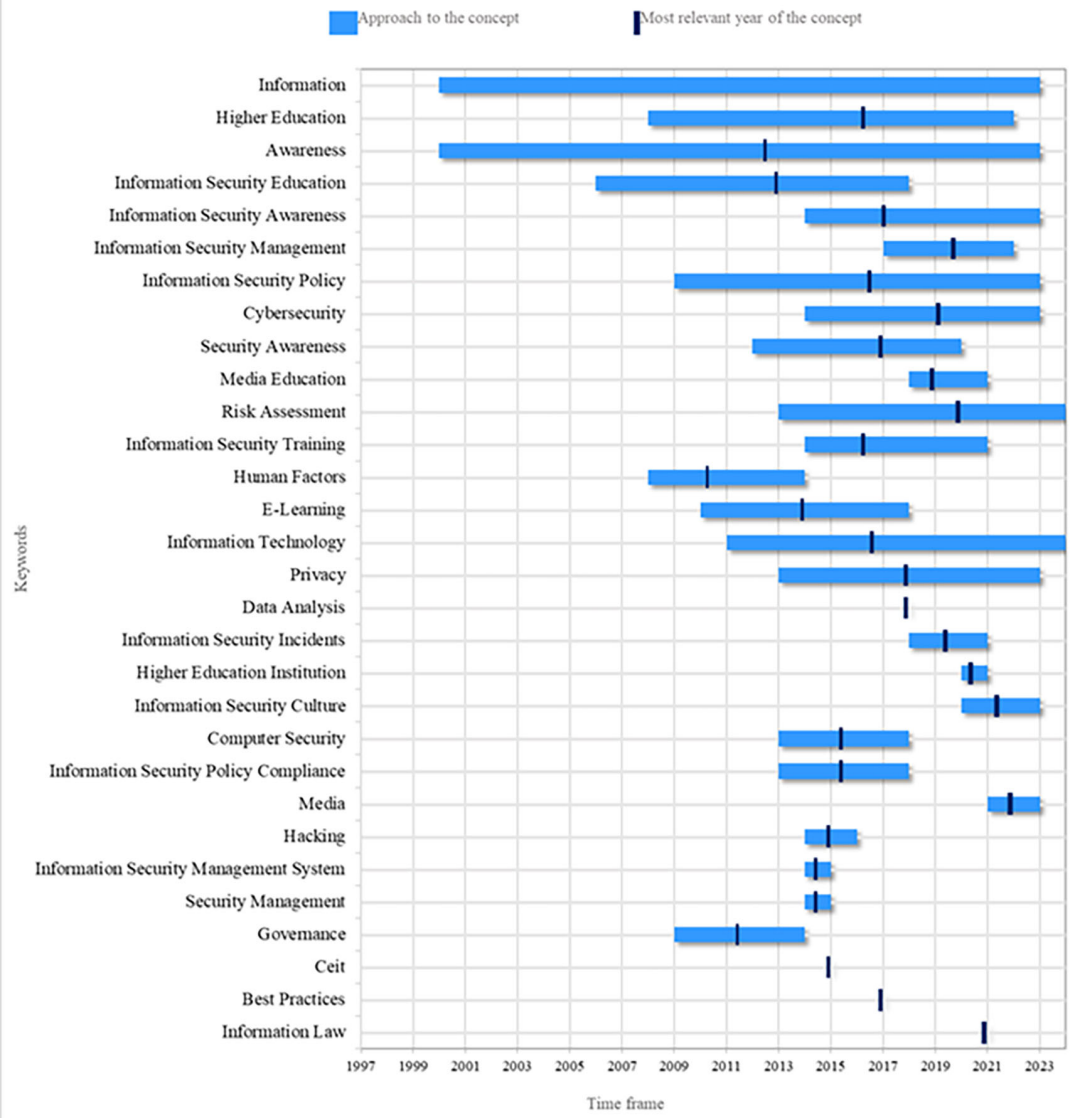
Research agenda. Own elaboration based on Scopus and Web of Science.

Finally, a research agenda is proposed with potential future lines of inquiry based on the findings of this study, as visualized in Figure 9. One of the topics with significant future potential is the culture of information security. Continuous failures in information security within organizations have highlighted the importance of focusing on organizational culture. It is argued that developing an information security culture would subsequently lead to a more secure organization; therefore, this topic should be considered in the education of information security professionals ( Tejay & Mohammed, 2023).

Similarly, the potential of media is noteworthy, as it facilitates education in information security. Consequently, research has been developed to understand the current challenges in information literacy, analyze their causes, and offer solutions and strategies. These studies provide valuable references for future information literacy education through various communication channels ( Tao, 2023). Additionally, the relevance of information regulations is highlighted. Information technology, as a regulated element, is interpreted differently across various sectors of security legislation. Therefore, research in this field is essential to promote consistency in the regulatory framework ( Brinker, 2024).

Higher education institutions also emerge as a relevant topic for future research on compliance with information security policies. Although the literature in this area is still limited, it shows promising advancements ( Hina & Dominic, 2020). Another crucial aspect is information security risk assessment, which is a fundamental component of business management practices. This assessment helps to identify, quantify, and prioritize risks based on risk acceptance criteria and organization-specific objectives. Therefore, education in this area is essential for information security professionals ( Kuzminykh et al., 2021).

Information security management is a critical issue that must be continuously addressed in the future. As Merchan-Lima et al. ( Merchan-Lima et al., 2021) emphasize, adopting effective information security management practices is imperative, especially in the context of higher education. Future professionals should be trained to design and implement these practices. Additionally, cybersecurity plays a significant role in the future of higher education. According to Kweon et al. ( Kweon et al., 2021), security training and education are effective methods for detecting cyberattacks in both academic and industrial settings. Their research found a positive association between cybersecurity training and a reduction in cyberattacks within organizations.

Based on the identified potential topics, the following research questions are proposed for future research:

• What are the main aspects of information security culture in organizations, and how do they influence the effectiveness of training programs?
• What role does the media play in promoting information security awareness and education among the general public and industry professionals?
• What are the most relevant international and national (e.g., Colombia) information security regulations, and how do they impact information security education programs?
• How are higher education institutions integrating information security into their academic programs, and what pedagogical approaches are they using?
• What are the key challenges in managing information security within organizations, and how can these challenges be addressed through education and staff training?
• What are the essential competencies that information security professionals need to acquire, and how are these competencies being developed in education programs?
• What strategies are organizations implementing to foster a culture of cybersecurity among their employees, and how are they evaluating the effectiveness of these strategies?
• How are higher education institutions collaborating with industry and other organizations to ensure that information security training programs align with labor market needs?
• What are the emerging trends in cybersecurity, and how are they being addressed in education programs to ensure relevance and up-to-date knowledge?
• What is the impact of information security education on risk reduction and data protection within organizations and society at large?

## 4. Discussion

Information security education and awareness are crucial issues in today’s world. Similar to the study conducted by Amankwa et al. ( Amankwa et al., 2015), a literature review has been employed to evaluate models aimed at improving security education and awareness. These models focus on stakeholder domains such as end users, institutions, and industry. This study also identifies a research gap regarding the relationship between higher education institutions and industry (a key stakeholder), as well as other organizations, to ensure that information security education programs align with labor market needs.

Previous research aligns with the findings of this study on information security policies. For example, Alassaf and Alkhalifah ( Alassaf & Alkhalifah, 2021) found that the literature lacks information on the positive and negative (direct or indirect) influences of human and organizational theories and their factors affecting information security policy compliance behavior. This study also identifies gaps in higher education related to information security policy education. Addressing these gaps is crucial to ensure that future professionals are prepared to design and implement secure practices within higher education institutions.

The results of this study are consistent with insights from other research, such as the systematic review by Hina and Dominic (2020) on compliance with information security policies. Their review emphasized that limited attention has been given to critical infrastructures, including higher education institutions, that are particularly vulnerable. They also examined the role of institutional governance in shaping protection motivation for information security. Similarly, this study underscores the importance of further exploring the intersection of information security policies and higher education. It also highlights the need for information security professionals to develop essential skills in policy implementation and gain a deeper understanding of how these policies are integrated into educational programs.

The findings of the study by Merchan-Lima et al. ( Merchan-Lima et al., 2021), who conducted a systematic literature review on effective information security management practices in higher education institutions, align with the results of this research. These authors suggest that future research should focus on how to educate users and the mechanisms to help higher education institutions select the appropriate risk assessment models for their needs. This study similarly highlights the importance of information security management and risk assessment within the context of professional education, underscoring the need for further exploration in future studies. Additionally, it emphasizes the relevance of designing curricula that align with the current needs of organizations.

This study contributes from two fundamental perspectives. First, from a theoretical standpoint, it identifies the predominant trends in information security education research. Additionally, it explores emerging themes, underdeveloped areas, and persistent challenges. These findings provide a clearer picture of the structure of knowledge in the field of information security education, including the relationships between influential concepts, authors, journals, and countries. Second, the study highlights underexplored areas and topics that require further attention in future research. This information is valuable for formulating new hypotheses and raising relevant research questions. Ultimately, it contributes to the advancement and enhancement of information security practices in an increasingly digitized world.

In terms of practical implications, the findings of this research may be valuable as a guide and foundation for planning higher education training programs. These results offer relevant information on key topics, methods, and approaches in the field of information security, which may influence the design of academic and training programs.

In addition, these findings can support decision-making in both academia and business. They help to understand the needs in information security education and to allocate resources more effectively. By examining the existing literature, best practices in information security education based on currently relevant topics have been identified. This information can serve as a reference for the development and implementation of successful programs.

Additionally, evidence gathered from existing literature on the impact of information security education interventions, such as awareness programs or professional development initiatives, is valuable for informing future strategies. Finally, the findings of this study can be used as input for designing international benchmarking and comparison reports. By comparing scientific output in information security education across different countries, institutions, and sectors, opportunities for collaboration and mutual learning can be identified ( Escuela Europea de Excelencia, 2021).

Similarly, the study has some limitations. First, it is based on the Scopus and Web of Science databases, which are the most widely used in academia. However, it would be beneficial to explore other databases, such as IEEE, that focus on engineering topics, including information security. Second, the selection criteria were not extremely specific, so future research could define more detailed criteria and carry out a systematic literature review to deepen the research topic. Third, although the study analyzes thematic trends, it does not focus as much on research gaps. Despite this, a research agenda is provided to guide future studies.

The following Table ( Table 1) presents a summary of the gaps identified in the research, these gaps cover various aspects, from the integration of information security in academic programs to the lack of standardized assessment methodologies and the need for an interdisciplinary perspective, each gap is accompanied by a brief justification and questions to guide future research in the field, this table provides an overview of the critical areas that require attention and development to advance information security education.

**Table 1.** Convergent validity testing results.

Gap type	Gap	Justification	Questions for future research
Methodological	Shortage of research on the integration of information security in higher education academic programs.	Although the importance of integrating information security into academic programs is recognized, there is a lack of specific research on how this integration is being done and what are the most effective approaches.	- How are higher education institutions integrating information security into their academic programs and what pedagogical approaches are they using? - What are the best practices for integrating information security into academic programs?
	Gap on the impact of information security education on risk reduction and data protection in organizations and society at large.	Despite the growing importance of information security, there is a lack of research on the concrete impact of information security education on risk reduction and data protection.	- What is the impact of information security education on risk reduction and data protection in organizations and society at large? - What education strategies are most effective in achieving these objectives?
	Lack of standardized methodologies to evaluate the effectiveness of information security education programs.	Although various information security education programs have been developed, the lack of standardized methods for evaluating their effectiveness makes it difficult to compare different programs and identify best practices.	- What are the most effective methodologies for evaluating the effectiveness of information security education programs? - How can researchers develop standardized methodologies for evaluating the effectiveness of education programs?
Data and Metrics	Shortage of available data and metrics to measure the impact of information security education programs.	The lack of specific data and metrics makes it difficult to accurately assess the impact of information security education programs, limiting our understanding of their effectiveness and ability to improve them.	- What data and metrics are most relevant to measuring the impact of information security education programs? - How can these data and metrics be effectively collected and analyzed?
Interdisciplinary	Need for an interdisciplinary perspective to address information security challenges from multiple angles.	Given the complex and multifaceted nature of information security challenges, an interdisciplinary perspective that integrates knowledge from different fields, such as technology, psychology, and politics, is required to develop effective solutions.	- How can interdisciplinary approaches improve the understanding and management of information security risks? - How can researchers from different disciplines collaborate to address information security challenges?

Integrating information security into higher education programs is crucial for ensuring that students are equipped with the necessary skills to address modern cyber threats. This integration helps bridge the gap between theoretical knowledge and practical application, preparing graduates to handle real-world security challenges effectively. Developing detailed curricula that include up-to-date practices and emerging technologies could greatly enhance students’ readiness for the evolving landscape of information security.

Another significant gap lies in the lack of standardized methodologies for evaluating the effectiveness of information security training programs, without a consistent evaluation framework, it is difficult to determine which pedagogical approaches are most effective and how to improve the quality of the training offered, this gap highlights the need to develop robust evaluation tools that can measure the real impact of information security training on risk reduction and data protection.

Furthermore, the paucity of data and metrics available to measure the impact of information security education programs presents an additional challenge. The lack of quantitative and qualitative information limits our understanding of the effectiveness of these programs and makes it difficult to identify areas for improvement, it is critical to address this gap by systematically collecting relevant data and developing robust metrics that can guide informed decision-making in the design and implementation of information security education programs.

Your analysis highlights crucial areas where further research and development are needed. Addressing these gaps can significantly enhance the effectiveness and relevance of information security education programs. By focusing on integration, standardized evaluation, data collection, and interdisciplinary approaches, future research can contribute to a more comprehensive and robust framework for preparing professionals to tackle the evolving challenges in information security.

## 5. Conclusion

The increasing complexity and frequency of cyber threats underscore the critical need for robust information security education. Universities play a pivotal role in this by preparing future professionals with the necessary skills and knowledge to combat these threats effectively. As technology evolves and cyber risks become more sophisticated, higher education institutions must adapt their programs to ensure they align with current and future demands. This includes not only teaching technical skills but also fostering a deep understanding of organizational policies, risk management, and the broader context of information security.

The objective of this research was to identify thematic trends in the field of information security education. To achieve this, five research questions were formulated. The first question made it possible to identify the research actors, i.e., the most influential authors, journals, and countries in this field. Authors Von Solms R, Safa NS, and Furnell S, as well as Rezgui Y and Marks A, stand out as the most influential due to their large number of publications and citations recorded. In terms of journals, Computers & Security stands out as the most influential, with a high volume of publications and citations on this topic. In addition, the United States and South Africa are the most relevant countries in this context. This leads to the conclusion that the aforementioned actors are the ones that generate the most impact within the field of education research in information security.

The second research question focused on the thematic evolution in recent years within the research field. Through an analysis of the most recurring keywords for each year, significant researcher interest was identified in the following topics: security perception, higher education, information technology, security behavior, data protection, and data security. The third research question addressed thematic research clusters. In this analysis, the following information security relevant clusters were identified: information security education, information security awareness, higher education, information security management, cybersecurity, and information security training.

In the fourth research question, an analysis of the currency and frequency of keywords related to the research topic was conducted. The findings revealed that the most frequent and current topics are information security awareness and higher education. This suggests that future research can be oriented towards these two topics. Finally, from the fifth question, a research agenda was proposed that identifies potential in the following thematic areas: information security culture, media, information security regulations, higher education institutions, information security management, and cybersecurity. From these thematic areas, research questions were proposed that could be answered in future research.

## Ethics and consent

Ethical approval and consent were not required.

**Disclosure statement.** No.

## Data Availability

No data are associated with this article. Zenodo: Dataset and supporting materials for the study “Information security education: a thematic trend analysis”. https://doi.org/10.5281/zenodo.14271117 ( Valencia-Arias, 2024). The project contains the following underlying data:
1. PRISMA Flow Diagram (Flowchart illustrating the study selection process based on PRISMA guidelines).Dataset.xlsm (Raw data supporting the findings of this study). 2. Editable _ seguridad de la info en educación.xlsm 3. PRISMA Checklist.pdf (Checklist detailing compliance with PRISMA 2020 guidelines). Data are available under the terms of the Creative Commons Attribution 4.0 International license (CC-BY 4.0).
