# Supplementary material for: Information security education: a thematic trend analysis
Source: F1000Res. 2025 Feb 17;14:5. Originally published 2025 Jan 2. [Version 2] doi: 10.12688/f1000research.159828.2 (PMC11982802; doi:10.12688/f1000research.159828.2)
Supplement: Peer review history

# Reviewer report: Reviewer response for version 2

**DOI:** 10.5256/f1000research.178018.r372462

**Published:** 2025-04-09

**Authors:** Arina Alexei

**Affiliations:** 1 Technical University of Moldova, Moldova, Moldova

I believe that the content of the article is appropriate. The findings obtained through the review of the specialized literature are both interesting and informative. The article effectively highlights current and future research directions, which is important for understanding the development of the field. The review methodology is clearly explained, making it replicable for other researchers. The statistical analysis is well-structured and adequately supports the research questions in a logical and argumentative manner. The conclusion is well defined; it briefly summarizes the research findings and also discusses the limitations, which helps readers understand the context of the study. These limitations could serve as inspiration for other researchers to further explore the topic.

 My only remark is that the reviewed literature is not predominantly from recent years. Considering that the field of information security is highly dynamic and subject to rapid changes, it would be beneficial for educational approaches to focus more on recent trends.

Are the rationale for, and objectives of, the Systematic Review clearly stated?

Yes

Is the statistical analysis and its interpretation appropriate?

Yes

If this is a Living Systematic Review, is the ‘living’ method appropriate and is the search schedule clearly defined and justified? (‘Living Systematic Review’ or a variation of this term should be included in the title.)

Yes

Are sufficient details of the methods and analysis provided to allow replication by others?

Yes

Are the conclusions drawn adequately supported by the results presented in the review?

Yes

Reviewer Expertise:

My research interests lie in information and cyber security across public and private organizations of various sizes, with particular emphasis on the healthcare and education sectors.

I confirm that I have read this submission and believe that I have an appropriate level of expertise to confirm that it is of an acceptable scientific standard.

# Reviewer report: Reviewer response for version 2

**DOI:** 10.5256/f1000research.178018.r367030

**Published:** 2025-02-25

**Authors:** Lynette Drevin

**Affiliations:** 1 North West University, Potchefstroom, South Africa

Thank you. 

 I do not have any other comments.

Are the rationale for, and objectives of, the Systematic Review clearly stated?

Yes

Is the statistical analysis and its interpretation appropriate?

Not applicable

If this is a Living Systematic Review, is the ‘living’ method appropriate and is the search schedule clearly defined and justified? (‘Living Systematic Review’ or a variation of this term should be included in the title.)

Yes

Are sufficient details of the methods and analysis provided to allow replication by others?

Partly

Are the conclusions drawn adequately supported by the results presented in the review?

Yes

Reviewer Expertise:

Human aspects of IT, Information security & awareness

I confirm that I have read this submission and believe that I have an appropriate level of expertise to confirm that it is of an acceptable scientific standard.

# Reviewer report: Reviewer response for version 1

**DOI:** 10.5256/f1000research.175613.r356007

**Published:** 2025-01-25

**Authors:** Lynette Drevin

**Affiliations:** 1 North West University, Potchefstroom, South Africa

1 Are the rationale for, and objectives of, the Systematic Review clearly stated?

 The aim of the paper is presented and the research questions are stated clearly and are addressed in the paper.

 2 Research design and methods:

- Adherence to the PRISMA reporting guidelines – Yes
- Inclusion criteria – This is stated. However – the terms awareness and human behaviour in information security would have been valuable in the searches.
- Information sources -  A few of the sources is quite old in the context of this fast-evolving field. Examples will follow at the end of this report.
- Search strategies – it is confusing to the reviewer why certain sources – such as conference proceedings are left out in the search. It is important to have recent studies – in contrast of the reason given in section 2.3 to leave those out.

For example – IFIP WISE (World Conference on Information Security Education) has Scopus  outputs that could be valuable.

https://link.springer.com/conference/wise-ifip

 As could HIASA be -  IFIP International Symposium on Human Aspects of Information Security & Assurance. https://haisa.org/

 Scopus outputs: https://link.springer.com/conference/haisa

 3 Are the conclusions drawn adequately supported by the results presented in the review? Yes, mostly – but in section 3.5 where future work is proposed – the authors could have searched what is already being done and addressed on their topics identified. E.g. bullet 4 is addressed in papers and work done by IFIP WG11.8 (WISE) as stated above. Also see . https://cybered.hosting.acm.org/wp/

 ACM/IEEE/AIS SIGSEC/IFIP Cybersecurity Curricular Guidelines  CSEC.

 There is also a list of prominent academics and researchers that worked on this – and may be added to the names of people involved in these studies.

 The other bullets may also already have work done on the topics stated. This could have been searched and acknowledged.

 Figure 3- Most relevant authors: As certain important literature was excluded; some important authors were not part of the results – e.g. Prof Matt Bishop in the USA. Prof LA Futcher in South Africa, etc.

 Regarding the names stated in figure 3 as most relevant authors – the reviewer was hoping to see more of their work cited in the reference list. E.g. Von Solms, Furnell etc.

 Future work is stated.

 4 General remarks

 Page 7 – Do not use the word below when referring to a table of figure. Below (See Figure 5) – and then the figure is not below but in the top of the page 

 Where more or more recent sources are needed:

 Section 1 – par 2 – Rocha Flores W, et al., 2016 (Ref 1)

 Par 5 Cheung et al 2011 – newer source where universities are of concern – see how the ACM/IEEE CSEC guidelines were developed. See Futcher on curriculum issues.

 Section 3.1 Par 4 Rezgui & Marks – 2008. Maybe more sources can be added to strengthen this topic of researchers: 

 See:  for example,

 - Bada M, et al., 2019 (Ref 2)

 Kruger H, et al., 2010 (Ref 3)

 Drevin L, et al., 2007 (Ref 4)

 Section 3.3

 Par 2 Gjertsen et al 2017 –  add more recent sources on gamification and security e.g.

 -  Silic M, et al., 2020 (Ref 5)

 Thank you for an interesting article.

Are the rationale for, and objectives of, the Systematic Review clearly stated?

Yes

Is the statistical analysis and its interpretation appropriate?

Not applicable

If this is a Living Systematic Review, is the ‘living’ method appropriate and is the search schedule clearly defined and justified? (‘Living Systematic Review’ or a variation of this term should be included in the title.)

Yes

Are sufficient details of the methods and analysis provided to allow replication by others?

Partly

Are the conclusions drawn adequately supported by the results presented in the review?

Yes

Reviewer Expertise:

Human aspects of IT, Information security & awareness

I confirm that I have read this submission and believe that I have an appropriate level of expertise to confirm that it is of an acceptable scientific standard, however I have significant reservations, as outlined above.

## References

[1] : Shaping intention to resist social engineering through transformational leadership, information security culture and awareness. *Computers & Security* .2016;59: 10.1016/j.cose.2016.01.004 26-44 10.1016/j.cose.2016.01.004

[2] : Developing cybersecurity education and awareness programmes for small- and medium-sized enterprises (SMEs). *Information & Computer Security* .2019;27(3) : 10.1108/ICS-07-2018-0080 393-410 10.1108/ICS-07-2018-0080

[3] : A vocabulary test to assess information security awareness. *Information Management & Computer Security* .2010;18(5) : 10.1108/09685221011095236 316-327 10.1108/09685221011095236

[4] : Value-focused assessment of ICT security awareness in an academic environment. *Computers & Security* .2007;26(1) : 10.1016/j.cose.2006.10.006 36-43 10.1016/j.cose.2006.10.006

[5] : Using Design-Science Based Gamification to Improve Organizational Security Training and Compliance. *Journal of Management Information Systems* .2020;37(1) : 10.1080/07421222.2019.1705512 129-161 10.1080/07421222.2019.1705512

# Response

**Published:** 2025-02-05

**Authors:** JHOANY ALEJANDRO VALENCIA ARIAS

In accordance with the suggestions of the reviewer in our article **“Information security education: a thematic trend analysis”**, the following changes were made, properly marked with red letters in the article:

**Comment 1**

 Inclusion criteria – This is stated. However – the terms awareness and human behavior in information security would have been valuable in the searches.

**Response 1**

 A paragraph of limitations on the inclusion criteria was added

**Comment 2**

 Information sources -  A few of the sources is quite old in the context of this fast-evolving field. Examples will follow at the end of this report.

**Response 2**

 The sources of information in the paper refer to the sources from which the articles necessary for the analysis were extracted, beyond the current nature of the conceptual and theoretical definitions

**Comment 3**

 Search strategies – it is confusing to the reviewer why certain sources – such as conference proceedings are left out in the search. It is important to have recent studies – in contrast of the reason given in section 2.3 to leave those out.

**Response 3**

 The exclusion of conference proceedings occurs because in many cases (not all), there is a lack of sufficiently structured and robust peer review processes, which could generate a bias in knowledge or less precise results

**Comment 4**

 in section 3.5 where future work is proposed – the authors could have searched what is already being done and addressed on their topics identified. E.g. bullet 4 is addressed in papers and work done by IFIP WG11.8 (WISE) as stated above. Also see . https://cybered.hosting.acm.org/wp/

 ACM/IEEE/AIS SIGSEC/IFIP Cybersecurity Curricular Guidelines  CSEC.

 There is also a list of prominent academics and researchers that worked on this – and may be added to the names of people involved in these studies.

 The other bullets may also already have work done on the topics stated. This could have been searched and acknowledged.

**Response 4**

 Information on the World Conference on Information Security Education (WISE) is included and the contribution of these scholars in cybersecurity education and the dissemination of knowledge, understanding and practice of computer security and information protection, as well as their influence on research agendas, is highlighted.

**Comment 5**

 Figure 3- Most relevant authors: As certain important literature was excluded; some important authors were not part of the results – e.g. Prof Matt Bishop in the USA. Prof LA Futcher in South Africa, etc.

**Response 5**

 The results are presented objectively in relation to our own findings in the databases. The absence of prominent authors within the results may be due to the fact that their research is not within the framework of the search strategy.

**Comment 6**

 Regarding the names stated in figure 3 as most relevant authors – the reviewer was hoping to see more of their work cited in the reference list. E.g. Von Solms, Furnell etc.

**Response 6**

**Four **more references from the main authors were included

**Comment 7**

 Page 7 – Do not use the word below when referring to a table of figure. Below (See Figure 5) – and then the figure is not below but in the top of the page

**Response 7**

 The expression, in accordance with the reviewer's recommendations, was deleted

**Comment 8**

 Where more or more recent sources are needed:

 Section 1 – par 2 – Rocha Flores W, et al., 2016 (Ref 1)

 Par 5 Cheung et al 2011 – newer source where universities are of concern – see how the ACM/IEEE CSEC guidelines were developed. See Futcher on curriculum issues.

 Section 3.1 Par 4 Rezgui & Marks – 2008. Maybe more sources can be added to strengthen this topic of researchers:

 See:  for example,

 - Bada M, et al., 2019 (Ref 2)

 Kruger H, et al., 2010 (Ref 3)

 Drevin L, et al., 2007 (Ref 4)

 Section 3.3

 Par 2 Gjertsen et al 2017 –  add more recent sources on gamification and security e.g.

 -  Silic M, et al., 2020 (Ref 5)

 1. Rocha Flores W, Ekstedt M: Shaping intention to resist social engineering through transformational leadership, information security culture and awareness. Computers & Security. 2016; 59: 26-44 Publisher Full Text

 2. Bada M, Nurse J: Developing cybersecurity education and awareness programmes for small- and medium-sized enterprises (SMEs). Information & Computer Security. 2019; 27 (3): 393-410 Publisher Full Text

 3. Kruger H, Drevin L, Steyn T: A vocabulary test to assess information security awareness. Information Management & Computer Security. 2010; 18 (5): 316-327 Publisher Full Text

 4. Drevin L, Kruger H, Steyn T: Value-focused assessment of ICT security awareness in an academic environment. Computers & Security. 2007; 26 (1): 36-43 Publisher Full Text

 5. Silic M, Lowry P: Using Design-Science Based Gamification to Improve Organizational Security Training and Compliance. Journal of Management Information Systems. 2020; 37 (1): 129-161 Publisher Full Text

**Response 8**

 Section 1. Paragraph 2. A more recent reference recommended by the reviewer was included. Section 3.1. Although the suggested references are relevant, they were not included, since they did not correspond to the authors mentioned in that section; however, other references from the main authors were included. Section 3.3. the suggested reference was included

 We look forward to your comments and hope to hear from you soon.

 Thank you very much.
